# Could walking football improve women’s quality of life throughout the menopause? An exploration of perimenopausal walking footballers in Scotland

**DOI:** 10.1186/s12905-025-04158-4

**Published:** 2025-12-11

**Authors:** Laura Wallace

**Affiliations:** University of the West of Scotland, University Avenue, Ayr, South Ayrshire, KA8 0SX Scotland, UK

**Keywords:** Menopause, Physical Activity, Women’s Health, Mid-life, Walking Football

## Abstract

**Background:**

Throughout their menopause journey many women experience symptoms that negatively impact their quality of life. In some cases, these symptoms can be alleviated with physical activity, however for many women, activity levels drop during this life stage. Aimed at women over 40, it is posited that walking football could be a useful intervention for improving quality of life in perimenopausal women.

**Methods:**

This is a qualitative study, exploring the experiences of women who play walking football, as they transition through their menopause journey. Semi structured interviews were conducted with seven perimenopausal walking footballers, before data was transcribed and thematically analysed.

**Results:**

Women experienced a range of symptoms, to varying degrees, that have a negative impact upon quality of life. Support from medical professionals was generally poor, and there was a lack of readily available information. Playing walking football provided both physical and mental health benefits, with the environment being highlighted as a ‘safe space’ for talking about menopause.

**Conclusions:**

Walking football may provide physical and mental health benefits throughout perimenopause and beyond. As a team sport, mental health benefits such as reduction in depressive symptoms, lower social isolation, higher self-efficacy, and improved body image can be further gained due to the camaraderie apparent among players.

**Supplementary Information:**

The online version contains supplementary material available at 10.1186/s12905-025-04158-4.

## Introduction

As women enter perimenopause, they can experience symptoms including hot flushes, sleep problems, vaginal dryness, low mood, brain fog, irritability and joint discomfort ([1–4]. Although there are exceptions, the National Institute for Health Care and Excellence (NICE) guidelines suggest otherwise healthy women aged 45 and over, who report any vasomotor symptoms, should be considered perimenopausal [5].

There is a plethora of evidence highlighting the positive impact of staying active through menopause, not only for general wellbeing, but also for the more specific impact on menopause symptoms [2, 6, 7]. The World Health Organisation (WHO) suggests that for general health, throughout each week adults should do at least 150–300 min of moderate physical activity (PA), 75–150 min of vigorous PA, or an equivalent combination [8]. However research suggests that, for many women, their PA levels drop during mid-life [9]. Reasons for this reduction in PA include time, family responsibilities, social support, and body image [10].

Women have reported a lower quality of life (QoL) through perimenopause, which is compounded by the reduction in PA, and menopause symptoms [4, 11]. As regular activity improves overall health, and QoL, this trend of decreasing participation with age in women is concerning. It is therefore important that middle aged women continue to be a focus of research, and practical interventions, that could lead to positive change.

Walking football (WF) is an inclusive sport. Originally aimed at older men (over 50), the sport sought to tackle inactivity, loneliness and social isolation [12]. This remains a key focus, however women over 40, and some younger people with health conditions or injuries, also enjoy participating. As well as being played at a slower pace, additional rules that disallow physical contact and heading the ball, support participation throughout the life cycle.

The youngest age category in women’s competitive WF is over 40, with a category for players aged over 50 [13]. This means most women who play will be reaching perimenopause, already perimenopausal, or post-menopausal. This makes players an ideal group in which to study the impact of activity on menopause symptoms. As many women report lower mental health (MH) throughout perimenopause [14], the additional social benefits gained from the unique nature of team sport, such as improved mood, body image, emotional self-efficacy, and general life satisfaction [15, 16], link well to participation in WF. Furthermore, as peer support is regarded a key support to women throughout their menopause journey [17, 18], participation in this team sport has the potential to have a wide-ranging positive impact.

To date there remains a lack of research into the impact of sport on menopause symptoms, especially in relation to team sports, with none specific to WF. The most common recommendation from health professionals is to take menopause hormone therapy (MHT), however not all women want to, or indeed can do this [19]. While many symptoms associated with perimenopause can be controlled, at least to an extent, through exercise, there remains a reluctance for exercise prescription from General Practitioners (GPs) [20]. WF is fast growing around the world, and currently has programmes for people with health conditions, such as Parkinson’s Disease [21] and Dementia [22]. This study therefore aims to investigate the potential impact of participating in the game, on perimenopausal women.

## Methods

### Participants

This study took place in the West of Scotland, UK, with female WF players. Inclusion criteria were: currently participating in WF, regardless of level/previous history of playing/etc.; experiencing at least one symptom associated with perimenopause. Use of MHT was not a determinant of inclusion in the study. Exclusion criteria were: women not playing WF currently, or previously while perimenopausal; women who had never experienced any menopause related symptoms. The sampling strategy was purposive, with recruitment done through the researcher’s personal contacts who acted as gatekeepers for their teams, and an advert on the Walking Football Scotland(WFS) website, and Facebook page.

### Research design

Following ethical approval from the researcher’s institution, WFS posted information on their website, linked on their Facebook page, explaining the study, and providing a contact email for anyone who may be interested. Gatekeepers sent this information direct to their contacts. Women who got in touch were sent a participant information sheet (PIS) and consent form, and offered the opportunity to ask any questions of the researcher. Once consent forms were returned, online interviews were arranged using Microsoft (MS) Teams, at a time convenient to the participant. To protect anonymity all participants were given a pseudonym.

These interviews were semi-structured; ensuring important topics were covered, while allowing for digression/elaboration where participants had interesting stories to share. See Appendix for a copy of the interview guide. The interview started with a general opener, to put the participant at ease, while gathering useful data about their history of PA and sport participation. Thereafter, questions focused on the menopause journey, symptoms experienced, treatment and support provided (or not), and experiences of playing WF. The interviewer focused on active listening, encouraging the participant to speak freely, interjecting only for clarification and prompting where more information about any points was sought.

Interviews lasted between 31 and 73 min (mean 39 min). These were audio and video recorded, allowing the researcher to revisit as codes evolved. MS Teams provided a transcription, however the researcher re-watched each interview upon completion, to make changes where words had been picked up wrong, largely due to local dialect. Following full transcription on MS word, each participant was sent a copy, to allow them to confirm it was an accurate representation of the conversation. This ‘member checking’ [23] helped triangulate the data, ensuring trustworthiness of the raw data. However as interpretative research is based on the ontological assumption that multiple realities exist, ‘member reflections’ [24] were also offered. Participants were emailed the transcription, and allowed to make any comments, or to add/remove anything they felt was no longer reflective of their situation. Upon completion of thematic analysis, a copy of the themes was also sent to the participants by email, and they were offered the opportunity to reflect upon these, and to engage in conversation with the researcher if they felt they had anything to discuss in relation to her interpretation.

### Positionality

It is of value to note the positionality of the researcher, due to the influence this can have on qualitative data collection and interpretation [25]. Although originally believed to be a binary of ‘insider’ or ‘outsider’ [26], more recently it has been argued that one’s positionality belongs on a continuum, along which the researcher may move with changing circumstances [27]. In this instance the researcher was towards the insider end of the continuum, as a female walking footballer who shared many physical characteristics, and experiences, with the participants. However, having not entered the perimenopausal stage of her life, she was not completely an insider.

The insider status brought benefits in line with those identified by Holmes [28], such as the ability to ask more insightful questions, gaining more honest and detailed discussion, and better understanding of language and non-verbal cues. Although these were key benefits, the researcher ensured positionality was acknowledged at the start of each interview, and that it was made clear that although some experiences were shared, that these experiences were individual, and not necessarily reflective of the participant’s experience of the same thing. Additionally, through regular reflexion [29], the researcher took a step back throughout the analysis of data, looking from different angles, to minimise any bias that insider status could bring.

### Data analysis

Following full transcription on MS word, Braun and Clarke’s [30] Reflexive Thematic Analysis (TA) was used to analyse the data. This followed the six steps of their original TA [31], including familiarising oneself with the data, generating initial codes, creating ‘candidate’ themes (that evolved through analysis), reviewing themes, creating a thematic map [32] and writing up findings. Coding was done primarily inductively, using NVivo, with avoidance of assumption based on previous experience. Key to inductive coding is the interpretation of verbal and non-verbal communication [31], which was enhanced by the revisiting of the interview videos and transcripts during the familiarisation process. This acknowledgement of the importance of language, and non-verbal communication, allowed rich data to be gathered.

## Findings

### Participant characteristics

The final sample consisted of seven women aged between 45 and 59 (mean 53), from four different WF clubs, all of whom were perimenopausal at the time of interview. The women ranged from never been ‘sporty’; through previously active, but had become less so with age; to those who had remained active throughout their lives. All women were white British. Although not representative of the general population, it is representative of those who currently play WF in Scotland (Table 1).

**Table 1:** Descriptive data for included participants

Participant (pseudonym)	Age	History of physical activity	Years playing WF
Claire	51	Played football informally in childhood, varied type/volume through adulthood	4
Jo	59	Played football informally in childhood, varied type/volume through adulthood	4
Michelle	50	Played football at primary school, giving up all sports at high school	3
Ally	52	Played organised football until injury in 20 s, continued with more individual/outdoor pursuits through adulthood	2
Gemma	54	Played sports (but not football) at primary school then sporadically ran, but little organised/regular sport	2
Kate	45	Played organised football until 20 s, fairly inactive until taking up WF	2
Jen	58	Played football informally in childhood, varied type/volume through adulthood	1

### Experiences of menopause and walking football

**Fig. 1:**
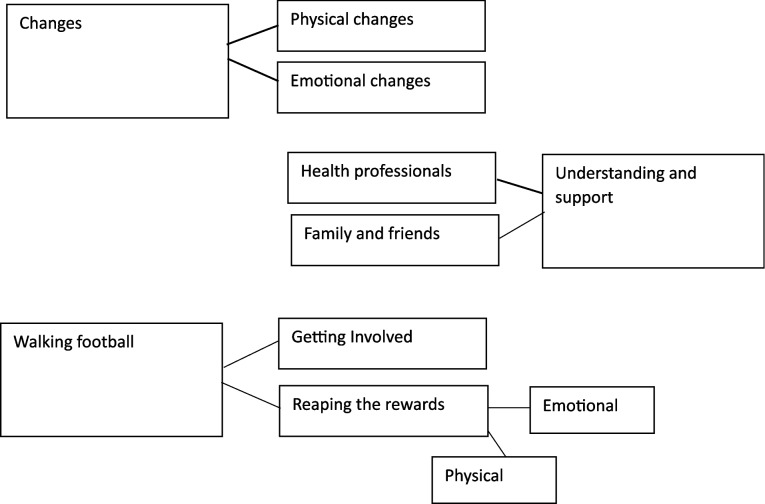
Thematic map

A total of 276 min of audio data was gathered, with the average interview lasting 39 min. Following an extensive coding process, where codes evolved/merged with each iteration, three overarching themes were generated. Related to each of these are two further themes, with one of these incorporating two sub-themes; presented in Fig. 1.

### Overview of themes

Changes explores the symptoms experienced by the women through perimenopause. Within the sub-themes of physical and emotional changes, the impact upon various aspects of their lives is analysed. Acknowledgement and Support relates to experiences of women as they reach, and negotiate, perimenopause. Sub-themes discuss the level to which others understood their position, and the support they received. Finally, Walking Football covers the women’s experiences of the game, from first becoming involved, and the ongoing impact it has had.

### Changes

All women had their own experiences of perimenopause, however they shared many commonalities. Most were in their mid to late forties when symptoms begun (*n* = 5), however two were still in their thirties. Symptoms negatively impacted upon a variety of aspects of their lives. Although severity of symptoms varied, for some it was, “*hell on earth*” (Jo, age59).

### Physical changes

The most widely reported physical changes were hot flushes and tiredness (*n* = 6), followed by weight gain (*n* = 4). Aching joints and heavy bleeding were occasionally reported (*n* = 2), with one woman experiencing severe vaginal dryness (*n* = 1).

Hot flushes were worst at night, but were experienced at all times during some periods. These were not only extremely uncomfortable, but they also impacted the women’s sleep (and that of their partners).

*Then the night sweats start. I didn’t have them through the day … I don’t always get them at night now, but when I do, it’s absolutely horrendous... you’re soaking like, 2–3 times a night. It’s so bad. It’s not just like, oh, wee sweat here and here. It’s so bad … you get so wet and then for some reason you start shivering like with the cold all of a sudden and, oh my god, I need to go and get changed again … Nightmare.* (Kate, age 45)

This interrupted sleep is likely to be a factor in the increased tiredness women felt. Tiredness and joint pain combined to negatively impact not only their general QoL, but also the amount and type of PA undertaken. Without exception, they stated that motivating themselves was difficult when physical symptoms were at their worst. Interestingly however, all reported increased activity levels since the onset of perimenopause. Although they felt tired, and sometimes sore, they were determined to overcome the symptoms, and to remain active, even when they did not feel like it.

*I’ve probably increased my exercise … You feel sluggish. Sometimes you don’t want to be bothered, you know. But I think I’ve become more active. Because you have to, have consciously made myself walk to the train … and that’s really helped me.* (Ally, age 52)

Their reasoning for doing this was that they were cognisant of the immediate, and long-term benefits of PA; this will be discussed later.

Although only mentioned by one woman, the impact of vaginal dryness is significant enough to warrant discussion as it had a marked influence on her relationship, and MH. Furthermore, as she had not been able to successfully have her regular cervical screening since the onset, this has the potential to impact her physical health. The woman spoke in depth about her relationship with her partner, over the long period of her ongoing perimenopause.

*I’ve not made love to him for seven year. Tried it once, maybe four year ago and I bled and it was so sore and I was like, no, you can’t do it. I just don’t want to. I went to see the doctor. I said 'I can’t live with this' because I miss that intimacy … I actually said to him [partner], ‘look listen, see if you want to go and find somebody, get somebody else', I says. 'I will totally understand that'.* (Jo, age 59)

Not only did Jo feel guilty not being able to give her partner the intimacy he desired, but she also missed it herself. At the time of interview she had had an on–off relationship with her partner, however in the member reflection she disclosed that the relationship was now over.

### Emotional changes

Emotional changes were more widely reported than physical ones, with brain fog and forgetfulness experienced by all participants (*n* = 7). Other changes included low mood (*n* = 6), anxiety (*n* = 5), and shortened temper/irritability (*n* = 4).

Irritability had a big impact upon family relationships among these women. Two discussed the *“craziness”* they experienced. For example, as well as impacting her relationship with her partner, Jo talked about the strain this put on her and her daughter’s relationship.

*I would come in from work and go,* [Raised voice] *‘why is that jacket..? Why? Why are you hanging your jacket over there? What? Why? You’re fucking hanging your jacket on the door…’ and then I would be banging doors … she decided she was going to go to uni…* (Jo, age 59)

Though not widely reported among this group, menopause symptoms, including brain fog and anxiety, did lead to some challenges at work.

*Just couldn’t concentrate. Struggled with work. A bit of, you know, real increase in anxiety. I used to be able to drive up and down the country. Got real anxiety about driving.* (Ally, age 52)

### Understanding and support

This theme covers the women’s journeys, from first experiencing symptoms, through coping with ongoing changes and challenges. There were rare mentions of understanding health professionals, however the more common narrative was that of dismissal. Family and peers were more often the source of emotional support, and sometimes helpful advice and signposting.

### Health professionals

With one exception, the women criticised the support provided by their GP. In relation to diagnoses, both who experienced symptoms before turning 40 were completely dismissed, being told they were too young. Both endured several futile visits, before being taken seriously. For one woman, the first time she felt her GP gave her symptoms serious consideration, was when her husband accompanied her to her appointment.

Upon diagnoses, most women were simply offered a prescription for MHT, with no discussion about other options. Some, however, despite being perimenopausal for at least two years, had not yet been offered any treatment options. Some women had visited numerous GPs in the hope of one understanding their symptoms. Interestingly, against the women’s expectations, it was often men who first took them seriously. The following quote illustrates the experiences of many.

*from the doctors and that, you don't get nothing, like absolutely nothing … even now like they just give me my pill and that's it … It’s like we’re just sort of left to get on with it. And I think that’s because I am younger … you think you would get more assistance from a female because obviously they know exactly what’s going to happen, or they’ve been through it, you know, but no, definitely guy, like the two guys that I have had have been a lot better.* (Kate, age 45)

### Family and friends

For those who had partners (*n* = 6), the general feeling was that were a good source of support. Those who had female partners (*n* = 2), had the additional benefit of not only empathy, but also a genuine understanding from experience.

*My partner said, ‘you need to get down the doctor and get patches' … She’s ahead of me, so yeah … sat me down and said 'get yourself down the doctor OK, go on the patches, that will help'. (Ally, age 52)*

Those with male partners (*n* = 4) also felt well supported, although their partners obviously could not directly relate.

*[partner]’s you know, he’s a good support, but [we] don’t really discuss it that much. (Claire, age 51)*

Some of the women had other female relations who they could turn to (*n* = 2), but those who did not felt particularly isolated.

*It’s not something you speak to your dad about*. (Jo 59)

### Walking football

Most women (*n* = 6) had played football, either formally or informally, when they were younger. For some this was only during breaks at primary school. However, regardless of history, they all saw taking it up in mid-life as an opportunity to have fun, in a slower paced game that they could enjoy while their bodies were less able than when they were younger.

### Getting involved

Those who had been active previously discussed how they had come to miss the sense achievement it led to, and the excitement of competition. For some, it was as their children grew up, that they found time to do something for themselves. For others, MH challenges were the primary factor in their decision.

Although some had played before, all felt extremely nervous attending their first session. However, they all spoke with passion about how welcoming the environment was.

I was quite nervous because I thought, oh God, you know, I’m not going to be able to play … I don’t really know anyone in the town because … I didn’t necessarily mix with other mums because I was working … so I was quite nervous, but they were really really welcoming. And everybody just, it was a laugh … It was supportive. It’s a supportive environment, which is nice. (Jen, age 58)

It was not only the words, but a latent analysis of the interviews found the feeling with which they were spoken, added to the meaning of this data. It was clear that the atmosphere in the WF community was key to women remaining involved. It is noteworthy that the welcome, and continued enjoyment, was unrelated to ability and experience. Both the experienced players, and complete novices, spoke with enthusiasm about how the sport caters for everyone, and that players in their own teams, and others, were nothing but supportive of everyone involved.

### Reaping the rewards

One overriding feeling was that, unlike previous attempts to be active, it was easy to be motivated for WF. As reluctance to get out and be active had been common among the women since entering perimenopause, this is significant.

*You know when you play football, you’re enjoying it. You’re not clock watching so often when you’re doing something like [that] … I’m not a member of a gym anymore, but that’s just because I have to force myself to go. And when I’m there I’m like, oh god, I’ve only been doing this for ten minutes … But you go to the walking football and that’s it … You are so absorbed … and it’s outdoors … you’re all doing the same thing.* (Jen, age 58)

Those who had previously been active were initially worried that, due to its nature, it may not be of a level to really improve their physical fitness, but they soon realised this was not the case.

*I’m getting that euphoric feeling back again because it’s active. People think ‘walking football, you’re just la la la la la...’ [but] no you’re not, you know. (Jen. Age 58)*

### Physical

Interviews showed significant physical health benefits from engaging in WF. Those who had joint pain found an improvement since starting to play, and more general fitness was widely improved, allowing the women to enjoy everyday tasks with more ease.

*We went to one of the festivals. And it was on a hill … I was able to run up the hill, and I thought ‘I wouldn’t have been able to do before’ … I just went for it. And I thought, ‘oh, that’s good because I wouldn’t’ have done that before and, obviously I’m getting a bit healthier’.* (Claire, age 51)

Furthermore, women generally felt less tired since starting to play WF.

*I think it has helped. I don’t feel quite so tired … that was one of my menopausal symptoms, the exhaustion … I don’t get nearly as tired now.* (Gemma, age 54)

A factor in this is likely the improved sleep many now experienced. Unsettled nights were widely discussed among symptoms, therefore this is a significant finding with potential to improve physical and mental health.

*I couldn’t sleep … They even gave me, think it was Zopiclone and that didn’t even work … I was just sitting up all night … and it wasn’t until I started going back to the football … and probably into the second or third week … I started to get a better sleep … I can sleep all night now without any medication. (Kate, age 45)*

### Emotional

Although physical benefits were discussed by all, it was evident that the biggest improvements were seen in MH. Despite ongoing symptoms continuing to negatively impact motivation to exercise, the women made the effort to play WF, knowing they would feel better both during and after.

*Sometimes I don’t want to go because I’m like, tired or, you know. But I always feel better after I’ve been … I feel so much better in myself … it’s not just the physical, it’s the social interaction … just feel better through physical exercise and also you know the highs and lows of sport*. (Ally, age 52)

Improved mood was noted by all, believed to be primarily a result of the distraction WF gave from the stresses of life.

*I enjoyed it because see that hour that you’re on the pitch, nothing else is going on in your head, so you get that break away from it, you know … you go along. You have that hour. You have a bit of a laugh. You think about the game you release some endorphins with the running about and then you come home and you’ve got that stories to talk about.* (Claire, age 51)

This improvement was emphasised through words, and the feeling behind them. Similarly, while women discussed their confidence levels dropping through perimenopause, they told of improvements since taking up WF, leading to wide reaching impacts beyond participation in the game.

*You feel more confident and you do more like there I was playing this morning and I’ll be playing with the recce girls tonight … I wouldn’t have considered going that in the past … before I started the walking football like, you get dead insecure about yourself [during perimenopause].* (Claire, age 51)

The findings above show the positive MH impacts upon all of these women through playing WF, however there are some significant examples worthy of note. The stories below are from players who disclosed diagnosed MH conditions, and ongoing challenges.

*[After my dad died] I went into a deep dark place. I really did nothing. Didn’t want to go out the door, was feart of my own shadow and everything, but getting back to football kind of changed that for me.* (Jo, age 59)

*It’s been a huge turning point for me. It’s been brilliant... I’m a lot better now … I always say it’s when I went back to the walking football, I always say that it saved my life.* (Kate, age 45)

Another key benefit discussed was peer support. Having had little from health professionals, this was valued very highly, not only for understanding and empathy, but also for valuable information.

*The biggest support just in knowing that other people are going through it is the walking football team … Because everyone’s very open and talks about stuff … my stepmum and mother-in-law don’t talk about it … my mum died a couple of years ago [but] she wouldn’t’ have talked about it … there is a certain amount of almost, shame about it. Maybe shame and denial … [But at walking football] people will post things on the group [chat] about like this and awareness groups, and yeah, people will just talk. Not huge big conversations, but there’ll be comments about menopause … I just know that that’s a space where talking about it is absolutely fine.* (Michelle, age 50)

*You can’t go to the doctors and they’ll be like, oh, this exists online or … they’ll give you all these numbers, but when you go especially when you’re younger, they just dismiss you … it’s just complete dismissal. I don’t think you get any support … it’s always like just medication or complete dismissal … and that sense of community, is worth more than any tablet they can give you.* (Kate, age 45)

In addition to the safe space for more serious discussions, WF also provided a platform for light-hearted discussions equally valued by women.

*You’ll discuss it with the girls at the football … you will have a bit of a moan about it, you know. But it’s like it’s more of a giggle at the same time. It’s like you know, I forgot to do this or I forgot to do that or, you know, just daft stuff*. (Claire, age 51)

*The football lot are good that way, they kind of just wee jokes and you know, talking about things and that’s supportive in its own way … comparing notes and talking through things*. (Ally, age 52)

## Discussion

The present study explored the journeys of perimenopausal women who currently play WF, seeking to find any associations between participation, and QoL. Seven women shared their stories of negotiating perimenopause where they felt less motivated to exercise, encountered poorer sleep, and found themselves to be irritable and forgetful. Upon taking up WF, all were made extremely welcome, regardless of ability and experience. Citing team camaraderie as key to their enjoyment and motivation, they discussed how the sport had impacted upon their QoL, in many domains.

With regards to symptoms, findings are in line with previous research. For example, in their survey of 947 perimenopausal women, Harper and colleagues [19] found most common symptoms to be mood swings, brain fog, fatigue, irregular periods, difficulty concentrating and night sweats. Similarly, Lu et al. [33], in their cross-sectional study of 295 Chinese women, reported insomnia, fatigue, bone and joint pain, sexual dysfunction and emotional instability as most common.

Experiencing symptoms is unfortunately inevitable for many, however the stories from the women in this study are worrying with regards to the support and guidance available. With only one sharing a positive experience, others found they had to work hard simply for symptoms to be acknowledged, and appropriate treatment offered. Although this sample was small, it appears reflective of the UK population. To investigate health care provision across the UK, Martin-Key et al. [34] gathered results from an online survey of 952 women, ranging from early perimenopause, to post-menopause. Here the majority (87%) found menopause symptoms to negatively affect their MH, however only 36% felt their health care provider took their symptoms seriously in relation to menopause, and only 24% were provided with any MH information.

Low mood is widely reported in many populations throughout perimenopause. Again, looking specifically at the UK, a recent systematic review of qualitative studies concluded, during menopause, women were more likely to experience MH challenges [35]. The overall consensus from the 32 included articles, with 3462 participants, also identified frustration about dismissal from doctors, and lack of sound advice in relation to treatment options. The experiences shared in the present study add backing to these previous findings, highlighting the importance of further research and action.

Highlighting that it is a more global issue, studies across the world show similar results. For example, Jia et al. [36] conducted a systematic review and meta-analysis of the global prevalence of depression in menopausal women. Although some key limitations were listed, it provides a reasonable account of women’s experiences, from 55 studies with 76,817 participants, across 16 nations. Statistical tests showed the prevalence of depression in perimenopausal women was 34%, compared to just 6% of the adult population [37]. It cannot be assumed that perimenopause is the sole cause, however the stories provided in the present study, combined with those published previously, suggest it likely plays a part, thus warranting further study and cognisance among health care professionals in the UK and beyond.

The importance of addressing risk of MH challenges cannot be underestimated. At the most extreme, depression and other mental illnesses can increase instances of suicidality. This is a significant concern among many populations, and specifically middle aged women. In their systematic review, Hendriks et al. [38] found that of 19 studies investigating suicidality among menopausal women, 16 reported positive associations. Many did not differentiate between menopause stages, however seven did find an association specifically in the perimenopause stage.

In studies of those who experienced menopause symptoms early (younger than age 45), instances of suicidality were further increased [39–41]. This specific demographic is less widely studied, however the present research adds some support to these findings. The two women who were not yet 45 upon first visiting their doctor with menopause symptoms, also disclosed MH issues. Although both had experienced depression prior, they did feel that their MH declined further during perimenopause. With the serious implications of declines in MH, and the delays in treatment associated with dismissal of symptoms, this is cause for concern.

Across the globe, women in general have lower levels of PA than men [42], especially in mid-life. A research report by Women in Sport [43] shows that 38% of women aged 45–54 do not achieve recommended levels of PA. This study involved an online survey and focus groups of inactive women aged between 45 and 60 in England. Survey findings showed 30% of respondents were ‘less active’ during menopause. Barriers to activity were categorised into three themes, capability barriers, opportunity barriers, and motivation barriers.

A more recent study of Irish women also found women to be less active during menopause, providing more evidence of the impact of menopause symptoms on PA levels [2]. Mirroring the findings of Women in Sport [43], results presented also identify physical symptoms and reduced perceived competence, (capability), lack of supportive environments (opportunity), and reduced energy and inclination (motivation).

Although women generally report lower levels of PA during menopause, it is not uncommon for them to state a desire to be more active. While those surveyed by Women in Sport [43] admitted to being less active during menopause, 80% said they would like to be more so. This finding was again mirrored by women in McNulty and colleagues’ [2] qualitative interviews. Like the women in the present study, it is apparent that women generally understand the benefits of being active, often leading to internal conflict between this desire to be active, and motivation to do so.

In the present study, although many discussed the challenges of menopause symptoms, and the effort it often took just to get out the house, they all had been active throughout perimenopause. This suggests that those who are inactive perhaps simply need an opportunity, and some encouragement. Ninety per cent of the women surveyed by Women in Sport [43] said they would consider PA if it was recommended by a health professional. This is a significant finding, and one which, if implemented, has the potential to impact women’s QoL, throughout menopause and beyond. When asked about treatment, there was no mention in the present study of the recommendation to be active. Some women were simply dismissed, some were offered antidepressants, and others prescribed MHT. None, however, felt that they were fully informed.

Among the sample in the present study were concerns about the potential increased risk of breast cancer, linked to MHT. These concerns have been documented previously [44], however more recent research [45] suggests the risk is low, and generally outweighed by the potential benefits. Current UK National Health Service (NHS) guidelines recommend taking MHT, with exceptions including those with an increased risk of breast cancer [46]. The increased risk made some women reluctant to start taking MHT, and tor those who did, they told how they were simply given a prescription and sent away, with no discourse.

All who were taking MHT spoke of how much it had improved their QoL, however it is also clear that their participation in WF has had a positive impact. With low mood being common among perimenopausal women, research about MH is of relevance. Many studies highlight the recommendation of prescribing PA, either instead of, or to supplement, medication [20, 47]. In the present study, the participant who had earlier disclosed suicidal thoughts, credited playing WF with her now improved MH. Evidence suggests that lack of knowledge acts as a barrier to some health professionals prescribing PA [20, 47]. It is posited therefore, that GPs should be better educated, allowing them to consider exercise prescription when women visit them, presenting menopause symptoms.

Specific to menopausal women, a scoping review by Hybholt [48] concluded that better MH outcomes were associated with higher PA during menopause. These findings were backed by a meta-analysis by Park and colleagues [49]. Added to the benefits of PA in general, team sports can provide additional benefits, resulting from the striving for a common goal, with people who have a similar interest [50]. In their longitudinal study based in Canada, Murray and colleagues [51], found that those who continued participation in team sport beyond high school, reported lower stress, and better coping, compared to non-participants. Similarly, in their systematic review, Eather and colleagues [52] found adults who participated in non-elite team sport, had more favourable health outcomes than those who participated in individual sport.

In many studies of coping strategies for menopause symptoms, peer support is highlighted as key [53–55]. This adds backing to the suggestion that team sport could be particularly beneficial to perimenopausal women. The findings in the current study provide valuable support to this. All appreciated, and at times relied upon, the support of their teammates, for both information and empathy. Camaraderie and peer support is key to team sport, and often the reason for long term participation.

In their interviews with adults in mid and later life, Gayman and colleagues [56] reported camaraderie and team spirit as integral to enjoyment, and thus long-term participation in team sport. Furthermore, in a recent review of articles published over a 37 year period, Thilakarathna and Chandana [57], highlighted many benefits of team sport participation among people with persistent depressive disorder. They also highlight team camaraderie, as well as the positive impact the social support in team sport can have on motivation, confidence and resilience. Although this study is not linked to menopause, key similarities can be found, adding some tentative evidence that WF could be promoted among perimenopausal women to help them manage some of their symptoms.

### Limitations

This study is limited in the small homogenous sample used. This means it is not representative of the general population, but is representative of those who currently play walking football in the West of Scotland. Also, through use of personal contacts for sampling, researcher bias cannot be ruled out. These personal contacts however, were generally stakeholders that recruited players unknown to the researcher, with only two of the women already known personally to the researcher. A reflexive diary was used throughout the data collection and analysis process, where the researcher reflected on each interview, looking at the data from different angles, to minimise any potential bias.

## Conclusion

This study illustrates the impact perimenopause can have on a woman’s life, resulting from a range of symptoms including brain fog, low mood, irritability and fatigue. Challenges are compounded by the lack of readily available information, options for controlling symptoms, and general understanding and support. The physical and social nature of WF led to wide ranging health benefits, including improved mood, better sleep, and improved fitness.

Although the study involved a limited sample, it provides novel findings that should be explored further. Future research with bigger samples, and over a longer time period, could further add to the evidence base, allowing for more sound recommendations for practice.

With many women wishing to be more active, especially during perimenopause, and the plethora of evidence showing the clear benefits of PA, the reluctance of health professionals to prescribe it more widely should be addressed. Through better education, GPs and other health professionals, could be better equipped to fully inform women of the various ways they can be supported through their whole menopause journey.

Furthermore, governing bodies, local councils, and health providers, should work to raise awareness of the game and its benefits, and consider offering specific sessions for perimenopausal women. A programme incorporating information and support sessions, and participation in WF would have the potential to lead to vast improvements in women’s menopause journeys.

## Supplementary Information

Supplementary Material 1.

## Data Availability

Full interview transcripts are held by the corresponding author, and may be available upon request.

## Abbreviations

FIWFA: Federation of International Walking Football Associations
GP: General Practitioner
MH: Mental Health
MHT: Menopausal Hormone Therapy
MS: Microsoft Teams
PA: Physical Activity
PIS: Participant Information Sheet
QoL: Quality of Life
SAMH: Scottish Action for Mental Health
WF: Walking Football
WFA: Walking Football Association
WFS: Walking Football Scotland
WHO: World Health Organisation
