# Supplementary material for: Could walking football improve women’s quality of life throughout the menopause? An exploration of perimenopausal walking footballers in Scotland
Source: BMC Womens Health. 2025 Dec 11;26:80. doi: 10.1186/s12905-025-04158-4 (PMC12882225; doi:10.1186/s12905-025-04158-4)
Supplement: Supplementary file 1 — Supplementary Material 1. [file 12905_2025_4158_MOESM1_ESM.docx]

**Appendix – Interview Schedule**

Please introduce yourself and tell me a bit about your engagement with physical activity throughout your life.

-prompt; early introduction, key influence, changes in levels, etc.

In as much detail as you are comfortable with, please explain your experience of the menopause.

-prompt; current stage, symptoms (current and past), changes, etc.

Do you think your physical activity levels have changed throughout your menopause journey? If so, how?

-prompt; reasons

Have you found any impact of physical activity – walking football or otherwise – on your symptoms (either positive or negative)? Explain.

Tell me about how you got involved in walking football. (If not already mentioned, ask about any previous football experience).

-prompt; initial knowledge of the game, early contact made, first / early sessions, etc.

How do you feel you’ve been supported through your menopause (consider friends, family, online / leaflet resources, GP professional bodies, etc.)?

Are there any ways you feel you could be / have been supported better to help you manage the impact of any symptoms?

If so, who by / how?

Discuss your involvement specifically with walking football, throughout your menopause. I’m interested to hear about any experiences, including how you’ve felt before, during and after.

Is there anything else you think may be of interest to me as I explore experience of female walking footballer as they go through their menopause?
